# Supplementary material for: Learning curve and surgical outcome of robotic assisted colorectal surgery with ERAS program
Source: Sci Rep. 2022 Nov 29;12:20566. doi: 10.1038/s41598-022-24665-w (PMC9709162; doi:10.1038/s41598-022-24665-w)
Supplement: Supplementary file 2 — Supplementary Information 2. [file 41598_2022_24665_MOESM2_ESM.zip › RAL Raw data-Ñ[▒K/061/OP N_ñw╝╨░O▒Kñσ.pdf]

W55 / W55

臺中榮民總醫院
手術室護理記錄

[redacted]

基本資料

- 室別： 開刀房-17室，手術科別： CRS，手術類別： 常規，日期： 2021/02/25
- 進入時間： 0730 ； 麻醉開始時間： 0750 ； Time Out時間： 0915 ； 劃刀時間： 0916 ； 手術結束時間： 1220 送出時間： 1245 ；
- 麻醉方式： GE
  診斷： Malignant tumor of sigmoid colon
  手術名稱： Robot Assisted Colectomy(L)
  手術局部用藥： NIL.
  術前預防性抗生素：
  - 抗生素名稱一： Veterin 1GM IVD由麻醉護理師執行
  - 時間： 0915
  - 上次簽章: 邱文琇

  ，追加預防性抗生素：
- 手術醫師： 蔣鋒帆，楊曙榮，林志安
- 麻醉醫師： VS 張思玲
-
  - 刷手護士A ： 廖子晴，時間： 0730，刷手護士B ： 羅媗鈴，時間： 0730，接替刷手一： 邱文琇，時間： 1130，接替刷手二： 邱文琇/廖子晴，時間： 1200
  - 流動護士A ： 邱文琇，時間： 0730，接替流動一： 羅媗鈴，時間： 1100，接替流動二： 蘇敬涵，時間： 1200

皮膚狀況

A-V Shunt： 無
術前： 完整
記錄者： 上次簽章: 邱文琇
術後： 完整
記錄者： 蘇敬涵

手術準備

手術臥式 單一手術臥式

-
  - 手術臥式： 截石臥式
  - 使用附件： 溫毯 43 ℃，頭圈，腳架，其它： lateral support
  - 皮膚準備： 10%Tr.Betadine，10%Aq Betadine，Hibiscrub
  - 電燒： 使用電燒: 電極片位置： 右大腿，雙極電燒，記錄者： 上次簽章: 邱文琇

止血帶T： 無

植入物

- 植入物名稱/號碼/數量/部位： Da Vinci Xi EndoWrist Stapler 45*1/S-colon

，記錄者： 蘇敬涵

標本送檢

- 細菌培養 無
- 細胞學 無
- 冰凍切片 無
- 病理標本
  組織名稱一： S-colon
  ,
  組織名稱二： IMA root
  ,
  組織名稱三： distal cut end
- 截肢 無

標本交付 標本： 3 袋，標本： 0 盒，標本： 0 瓶，細胞學： 0 管，Culture Tube： 0 管，被交付醫師： R 林志安，

- 記錄者： 蘇敬涵
- 時間： 1250

傷口術後敷料

傷口敷料： 傷口敷料： 無

- 請於圖上標示部位：

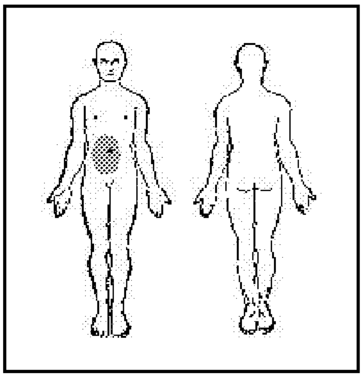

- 傷口敷料： 軟性敷料

導管

導管	天數	導管資訊	插入日期	插入時間	更換日期	拔除日期	拔除時間	拔除原因	預計拔除/更換	預計拔除/更換日期
Foley(1)	1	號碼:16 Fr.; 部位:膀胱;	2021/02/25	0850						
Double Lumen CVC(1)	1	號碼:8 Fr.; 部位:jugular (右); 象限:RU;	2021/02/25	0900						
Sump NG Tube(1)	1	號碼:16 Fr.; 部位:胃;	2021/02/25	0910						
A-line (1)	1	號碼:20 G; 部位:左手;	2021/02/25	0840						

用物計數

- 手術中出血量： Minimal c.c.
- 手術中尿量： 450 c.c.
-
  - 其它： 無

用物計數： 多切口同時縫合

- 1.器械： 正確，2.縫針： 正確，3.刀片： 正確，4.紗布： 正確，5.腹部墊： 未使用，6.紗球： 未使用，7.PEANUTS ： 未使用，8.紗條： 未使用，9.絲帶： 未使用，10.針頭： 未使用，11.Vessel loop： 未使用，12.Boots： 未使用，13.Jelco： 未使用，14.棉條： 未使用，
  - 第一次：
    - 刷手護士 ： 邱文琇
    - 流動護士： 上次簽章: 羅媗鈴
    - 時間： 1135

  ,
  - 第二次：
    - 刷手護士 ： 邱文琇
    - 流動護士： 上次簽章: 羅媗鈴
    - 時間： 1145

  ,
  - 第三次：
    - 刷手護士 ： 邱文琇
    - 流動護士： 蘇敬涵
    - 時間： 1200

備註

備註： Davinci scope NO.2
Olympus scope NO.1

- 記錄者： 上次簽章: 邱文琇
- 時間： 0730

填寫護理人員_蘇敬涵_______________

正式摘要

W55 / W55

doctorRank:B
